# Developmental Plasticity and Robustness of a Nematode Mouth-Form Polyphenism

**DOI:** 10.3389/fgene.2018.00382

**Published:** 2018-09-11

**Authors:** Bogdan Sieriebriennikov, Ralf J. Sommer

**Affiliations:** Max Planck Institute for Developmental Biology, Department of Integrative Evolutionary Biology, Tübingen, Germany

**Keywords:** *Pristionchus pacificus*, developmental plasticity, robustness, switch genes, Hsp chaperones, *Caenorhabditis elegans*

## Abstract

In the last decade, case studies in plants and animals provided increasing insight into the molecular mechanisms of developmental plasticity. When complemented with evolutionary and ecological analyses, these studies suggest that plasticity represents a mechanism facilitating adaptive change, increasing diversity and fostering the evolution of novelty. Here, we summarize genetic, molecular and evolutionary studies on developmental plasticity of feeding structures in nematodes, focusing on the model organism *Pristionchus pacificus* and its relatives. Like its famous cousin *Caenorhabditis elegans*, *P. pacificus* reproduces as a self-fertilizing hermaphrodite and can be cultured in the laboratory on *E. coli* indefinitely with a four-day generation time. However, in contrast to *C. elegans*, *Pristionchus* worms show more complex feeding structures in adaptation to their life history. *Pristionchus* nematodes live in the soil and are reliably found in association with scarab beetles, but only reproduce after the insects’ death. Insect carcasses usually exist only for a short time period and their turnover is partially unpredictable. Strikingly, *Pristionchus* worms can have two alternative mouth-forms; animals are either stenostomatous (St) with a single tooth resulting in strict bacterial feeding, or alternatively, they are eurystomatous (Eu) with two teeth allowing facultative predation. Laboratory-based studies revealed a regulatory network that controls the irreversible decision of individual worms to adopt the St or Eu form. These studies revealed that a developmental switch controls the mouth-form decision, confirming long-standing theory about the role of switch genes in developmental plasticity. Here, we describe the current understanding of *P. pacificus* mouth-form regulation. In contrast to plasticity, robustness describes the property of organisms to produce unchanged phenotypes despite environmental perturbations. While largely opposite in principle, the relationship between developmental plasticity and robustness has only rarely been tested in particular study systems. Based on a study of the Hsp90 chaperones in nematodes, we suggest that robustness and plasticity are indeed complementary concepts. Genetic switch networks regulating plasticity require robustness to produce reproducible responses to the multitude of environmental inputs and the phenotypic output requires robustness because the range of possible phenotypic outcomes is constrained. Thus, plasticity and robustness are actually not mutually exclusive, but rather complementary concepts.

## Introduction

Phenotypic plasticity is a feature of development whereby identical genotypes generate different phenotypes upon perception of environmental input (West-Eberhard, 2003). Examples of plasticity are ubiquitous and in extreme cases the alternative phenotypes produced are discrete, such as the various caste systems in social insects (Abouheif and Wray, 2002). However, the evolutionary significance of phenotypic plasticity is still widely debated. One view is that plasticity hampers evolution by enabling adaptation without genetic assimilation (Price et al., 2003). Conversely, the so called “flexible stem hypothesis” suggests a possibility that a phase of plasticity may be an obligatory step in the evolution of novel traits, whereby their expression remains conditional in the beginning before it becomes fully integrated into the development and fixed (Gibert, 2017). It is unknown in how many instances the origination of novel traits has followed this pattern, because careful phylogenetic studies of novel traits using the comparative method are scarce. Nonetheless, plasticity may increase evolutionary change through the simultaneous employment of multiple developmental pathways. Since every alternative pathway is only expressed in a fraction of the population or in a limited number of generations, selective constraints are relaxed and mutations can accumulate more quickly, thereby accelerating evolution (West-Eberhard, 2005; Susoy et al., 2015). Together, this makes phenotypic plasticity an important concept in both developmental and evolutionary biology.

Another fundamental feature of development is robustness, which is defined as the ability to generate identical phenotypes in the face of environmental perturbations and genetic variation (Wagner, 2005). Apart from the obvious role in maintaining the function of the organism under challenging conditions, robustness is argued to accelerate evolution by enabling accumulation of cryptic variation, which can be subsequently released and become material for selection (Rutherford and Lindquist, 1998; de Visser et al., 2003). Since the definition of plasticity entails sensitivity to the environment, whereas the definition of robustness entails insensitivity to it, the two phenomena are often contrasted. And yet, both plasticity and robustness have been suggested to accelerate evolution by releasing selective constraints – plasticity through conditional expression and robustness through concealing mutations from the forces of selection. This enigmatic relationship prompts the question if the two phenomena may, in fact, be complementary rather than opposing.

**FIGURE 1:**
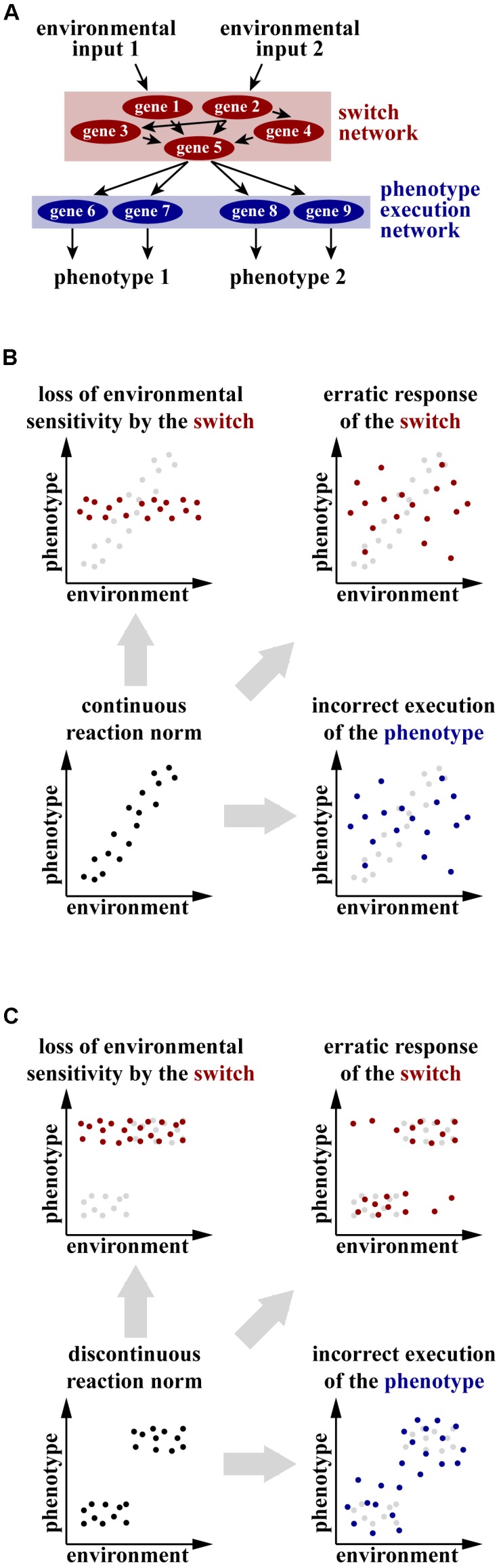
**(A)** Conceptual mechanism of the genetic regulation of plastic development. Environmental inputs are processed and integrated by a network of genes, referred to as switch genes. The switch network takes a decision to activate one of the alternative developmental programs and passes the signal down to a genetic network that executes the selected phenotype. **(B,C)** Hypothetical scenarios of change in the reaction norm of a continuously **(B)** or discontinuously **(C)** plastic trait in the conditions when the switch network or the phenotype execution network is impaired. The original distribution of phenotypes is shown in black in the foreground or gray in the background, altered distribution resulting from impairment of the switch network is shown in red and altered distribution resulting from incorrect execution of the phenotype is shown in blue.

Much of the discussion on the significance of plasticity and robustness for evolutionary change is based on theoretical arguments. In contrast, few experimental case studies address the molecular, genetic, and developmental mechanisms underlying these phenomena. Even more importantly, only a few experimental studies have investigated the potential crosstalk between plasticity and robustness simultaneously in the same study system. This is surprising as arguably knowledge about the mechanisms of plastic development can help address the interplay between plasticity and robustness in a more nuanced way than simply contrasting the two phenomena. The existence of a genetic control of plasticity and, therefore, the involvement of developmental switch gene networks and the execution gene network as separate developmental modules has long been debated (Bradshaw, 1965; Schlichting and Pigliucci, 1993; West-Eberhard, 2003). Developmental switches are genes that can change the developmental trajectory (Mather and de Winton, 1941; Ptashne et al., 1980). They do so by activating one or the other set of genes required for an alternative developmental pathway – sets, which we refer to as execution gene networks. For example, feminizing transcription factors *Sex-lethal* and *tra-1* act as developmental switches in *Drosophila* and *Caenorhabditis elegans*, respectively, because the level of their activity determines whether the animal will develop as a male or as a female/hermaphrodite. The targets of these switch genes are gene execution networks that generate traits typical of one or the other sex (Hodgkin, 1987; Bell et al., 1988). In this example, the developmental choice is mandatory and determined chromosomally, but studies on the genetic regulation of plasticity also confirmed the long-standing prediction about the involvement of switch genes in plastic development (for recent comprehensive reviews, see Fielenbach and Antebi, 2008; Projecto-Garcia et al., 2017; Sommer et al., 2017) (**Figure 1A**). Therefore, we suggest that the question of robustness of plastic traits can be addressed at two levels and that indeed robustness and plasticity are complementary concepts. First, genetic switch networks regulating plasticity require robustness to produce reproducible responses to the multitude of environmental inputs. Second, the phenotypic output requires robustness because the range of possible phenotypic outcomes is constrained. In the following we concentrate on a case study in a nematode, which explores the interplay between plasticity and robustness.

## Genetics of Plasticity: Continuous Vs. Discrete Phenotypes

In theory, the relationship between plasticity and robustness can be interrogated in any organism, however, some experimental systems possess features which greatly facilitate such studies. These features are, first, availability of isogenic lines, which simplify the genetics of the study system, and second, discreteness of alternative phenotypes. Specifically, studying genetically uniform individuals offers the possibility to unambiguously separate plasticity from polymorphisms generated by different genetic variants. As for the ability to generate discrete, as opposed to continuous, alternative phenotypes, such an ability potentially allows a sharper contrast between a constrained phenotypic distribution and conditions when developmental buffering is impaired and atypical phenotypes are produced.

More importantly, the hypothesis that genetic switch networks and phenotype execution networks are separate developmental modules whose robustness is provided by different mechanisms can only be explicitly tested in organisms in which impairment of the binary switches can be disentangled from expansion or displacement of the phenotypic distribution. Although a series of developmental switches is thought to underlie both continuous and discontinuous distributions of plastic phenotypes (West-Eberhard, 2003), phenotypic changes resulting from the manipulation of the switch and of the structural genes can be interpreted in different ways depending on the distribution of phenotypes. In the case of continuous traits, inactivation of genes channeling and integrating environmental inputs (the switch network) can either constrain the phenotypic distribution through a decrease in sensitivity to an inducing signal, or make it more variable as a consequence of improper integration of various environmental signals (**Figure 1B**). At the same time, tampering with the gene network executing the phenotype will also increase the variance of the phenotypic distribution (**Figure 1B**). Thus, developmental perturbations at the same level can potentially lead to different phenotypic outcomes, while manipulating different gene networks can lead to similar change. Together, this obscures the potential interplay between plasticity and robustness when continuously plastic traits are studied.

In contrast, manipulation of the switch and the execution networks will change the phenotypic distribution of discrete traits in a different manner. Interfering with the switch network will only affect the distribution of individual phenotypes *between* the discrete clusters, whereby the most extreme case would be the absence of individuals from some clusters (corresponding, e.g., to a loss of a morph). However, the distribution of phenotypes *within* the clusters is expected to be constant (**Figure 1C**). In contrast, loss of robustness of the gene network executing the phenotype is expected to affect the phenotypic distribution *within* the clusters (**Figure 1C**). Thus, only using organisms exhibiting discrete plasticity as a study model allows falsification of the hypothesis that plastic traits require robustness of both the switch and the execution gene network.

## The Study System: *Pristionchus pacificus* Mouth-Form Plasticity

**FIGURE 2:**
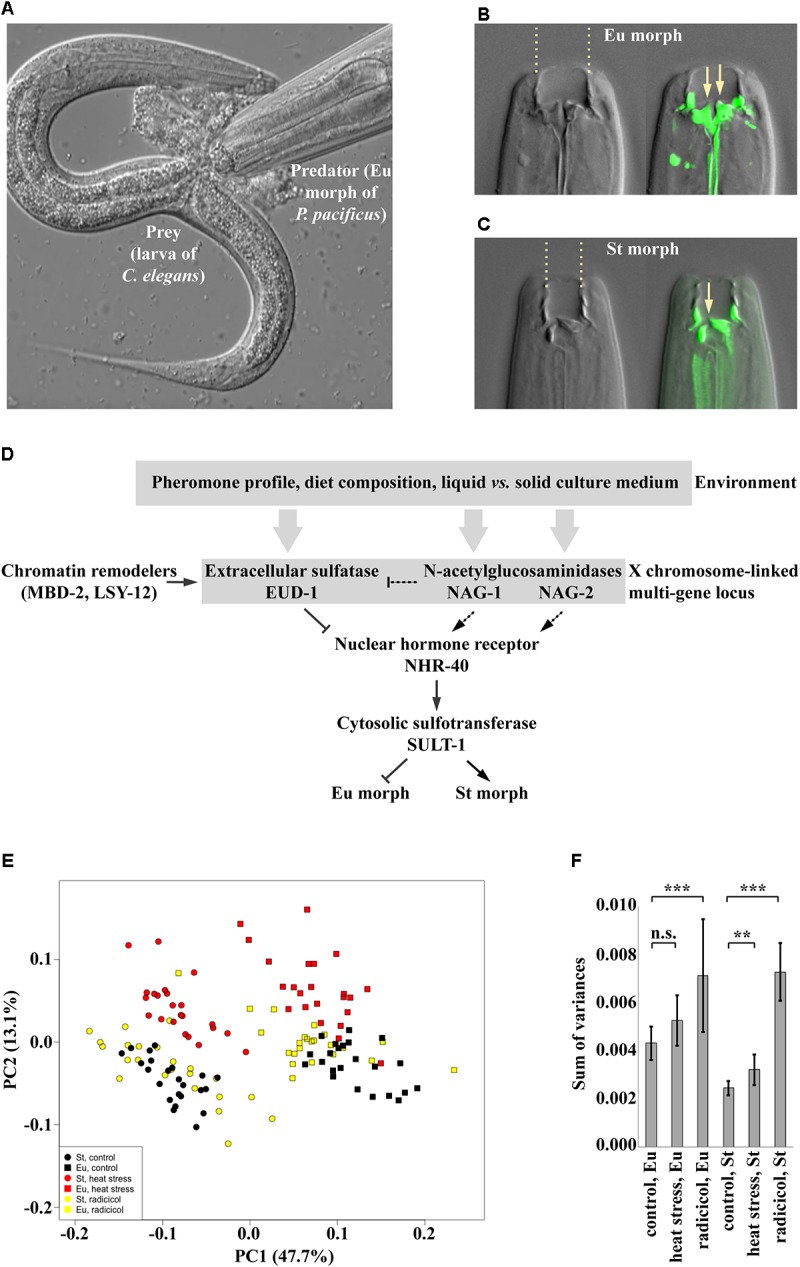
**(A)** The eurystomatous morph of *P. pacificus* devouring a larva of *C. elegans*. **(B,C)** The mouth of the eurystomatous **(B)** and of the stenostomatous **(C)** morph. On the left, differential interference contrast (DIC) image. On the right, overlay of the DIC image and an image of fluorescein-stained cuticle at the base of the buccal cavity, which includes teeth. Arrows point at the tips of teeth. **(D)** Current model of the regulation of mouth-form plasticity in *P. pacificus*. The genes shown are part of the switch network, i.e., mutations in these genes only change the frequencies of alternative phenotypes in the population. **(E,F)** Phenotypic effects caused by impairment of Hsp90 heat shock proteins, which are known to provide robustness to phenotype execution networks. In these conditions, both morphs are still produced but the morphologies are abnormal. **(E)** PCA ordination of sets of landmarks representing control individuals and individuals exposed to heat stress and treatment by radicicol, a pharmacological inhibitor of Hsp90. **(F)** Morphological disparity within different groups shown in the PCA ordination in panel **E**. Error bars show *SD*. n.s., not significant (*P*-value > 0.05); ^∗∗^*P*-value < 0.01; ^∗∗∗^*P*-value < 0.001. Panels E and F reproduced from Sieriebriennikov et al. (2017).

The nematode *Pristionchus pacificus* is a dimorphic species that belongs to the same order as the classical model *C. elegans* and shares its amenability to genetic manipulation, as well as the hermaphroditic mode of reproduction, which enables creation of isogenic lines (Sommer and McGaughran, 2013). Depending on the culture conditions, genetically identical individuals of *P. pacificus* can develop into two morphs – eurystomatous (Eu) (literally “wide-mouthed”) and stenostomatous (St) (“narrow-mouthed”) morphs. Eu animals possess a wide buccal cavity with two hooked teeth, which worms can use to kill other nematodes (**Figures 2A,B**). In contrast, the buccal cavity in St animals is narrow, the dorsal tooth is flint-shaped and the right ventrosublateral tooth is reduced to a cuticular ridge with a minute denticle (**Figure 2C**), which precludes killing, leaving such animals as obligatory microbial grazers (Bento et al., 2010; Wilecki et al., 2015). The decision on mouth-form development is taken during larval development and is irreversible in the adult stage (Serobyan et al., 2013). The developmental decision is influenced by the presence of pheromones, diet composition and the state of the culture medium (solid vs. liquid) (Bose et al., 2012; Sanghvi et al., 2016; Werner et al., 2017) (**Figure 2D**). Importantly, changing environmental conditions only alters the ratio between the phenotypes in populations, whereas intermorphs are extremely scarce. Together, these conditions make *P. pacificus* mouth-form plasticity an ideal study system to investigate the genetics, molecular biology and epigenetics of developmental plasticity and, building on the availability of such mechanistic insight, the potential relationship between plasticity and robustness.

Recent studies on the genetics of mouth-form plasticity in *P. pacificus* began to elucidate how the developmental decision is controlled. Forward and reverse genetic experiments implicated a locus on the X chromosome in switching between phenotypes (**Figure 2D**). This locus contains three functionally relevant genes, which are expressed in sensory and interneurons and which affect the phenotype ratio in the opposite manner. Specifically, the gene *eud-1* encodes an extracellular sulfatase and promotes the Eu morph, whereas *nag-1* and its paralog *nag-2* encode α-N-acetylgalactosaminidases, which additively promote the St morph (Ragsdale et al., 2013; Sieriebriennikov et al., 2018). Furthermore, the chromatin remodelers MBD-2, a methyl binding protein, and LSY-12, a histone acetyltransferase regulate *eud-1* levels (Serobyan et al., 2016; Serobyan and Sommer, 2017). The downstream transcription factor in the switch network is NHR-40, and the cytosolic sulfotransferase SULT-1 presumably acts downstream of *nhr-40* (Kieninger et al., 2016; Namdeo et al., 2018). Similar to manipulating environmental conditions, the manipulation of genes in this switch network only affects the ratio between the alternative morphs produced, but the morphologies of individual morphs remain intact. For example, *eud-1* mutant animals are all-St, whereas *nag-1 nag-2* double mutants are all-Eu. It is important to note that the current information on the genetic network is likely to be incomplete for several potential reasons. For example, genes that are part of the execution network might have essential functions earlier in development and as such, would go unnoticed in genetic screens as their phenotype would be lethal. Nonetheless, this genetic network for *P. pacificus* mouth-form plasticity provides a genetic and molecular platform for studying environmental influences, the evolution of plasticity and its relationship with robustness.

## Robustness of Developmental Switches

We suggested that plastic traits require the robustness of the switch network and of the network producing the phenotype (**Figure 1A**). The switch network integrates all the relevant environmental signals and takes the developmental decision, during which it faces several challenges. First, multiple contradictory environmental inputs can be perceived simultaneously. For example, the pheromone *dasc#1* induces the Eu morph in *P. pacificus*, whereas consumption of the yeast *Cryptococcus albidus* represses it (Bose et al., 2012; Sanghvi et al., 2016). Additionally, the developmental decision is often not taken instantaneously. Instead, the environmental signals accumulate over a prolonged time period, such as in *P. pacificus*, in which crowding has influence on the mouth-form ratio during all larval instars after hatching (Serobyan et al., 2013). Finally, development is inherently noisy and the precision of developmental decision-making processes, such as morphogen gradients, is generally limited (Gregor et al., 2007). Therefore, the ability to generate reproducible responses to multiple and potentially contradicting environmental inputs, while staying insensitive to noise, is crucial for developmental switches.

Such an ability is believed to be an intrinsic property of the architecture of gene regulatory networks (Masel and Siegal, 2009), with known examples in both vertebrates and invertebrates. For instance, the ecdysone receptor EcR in *Drosophila* positively regulates its own transcription and small fluctuations in ecdysone level or spontaneous transcriptional bursts could lead to a premature self-amplifying response (Herranz and Cohen, 2010). This is prevented by a negative feedback loop between EcR and microRNA miR-14. When the level of ecdysone is low, miR-14 represses the expression of the *EcR* gene, which ensures that EcR does not self-activate and remains poised for response to the elevated level of the hormone (Varghese and Cohen, 2007). An additional example is the circuit regulating neuronal subtype specification in mice in response to Sonic Hedgehog signaling, dissection of which revealed a network that consists of the transcription factors Olig2, Nkx2.2, and Pax6 connected with feedback and feedforward loops (Balaskas et al., 2012). Experiments with knockout lines and *in silico* modeling showed that negative feedback loops in the network provide robustness to small signal fluctuations, such that the network can respond to the morphogen by generating a highly reproducible pattern despite developmental noise (Gregor et al., 2007).

In *P. pacificus*, a phenomenon dependent on the robustness of the switch network during the regulation of mouth-form plasticity is the stochasticity of the phenotypic output. Although the phenotypic implementation of plasticity in this species is binary under normal circumstances, and as such intermediate morphs are extremely rare, there is apparent stochasticity as to which morph is finally adopted by an individual. The proportion of Eu morphs on agar plates under standard laboratory conditions fluctuates between 70 and 98%, even though all animals are genetically identical and grow in the same environment (Ragsdale et al., 2013; Werner et al., 2017). This pattern is consistent with the theoretical expectation of the phenotypic distribution produced by erratic action of the switch network (**Figure 1C**).

It is possible that such stochasticity is adaptive and represents a bet-hedging strategy – as the developmental decision is irreversible, it may be advantageous to always have some individuals of the underrepresented morph in the population in case the environmental conditions change more rapidly than a new generation can grow and develop suitable phenotypes (Losick and Desplan, 2008; Susoy and Sommer, 2016). In general, bet-hedging strategies are well-known in microbes that often face unpredictable environments (Veening et al., 2008). While the exact mechanism of how such stochasticity is generated is unknown, the gene regulatory network regulating plasticity may be susceptible to noise or it may even have a special mechanism to convert noise into phenotypic response. For example, cells forming sensory organ precursors in *Drosophila* are randomly selected from a field of equipotent cells due to noisy expression of the transcription factor Senseless, followed by lateral inhibition of the neighboring cells (Jafar-Nejad et al., 2003). In such cases, sensitivity to developmental noise may be advantageous for the organism.

The alternative view is that the response of the gene network is robust to noise and the apparent stochasticity in the phenotypic outcome results from environmental micro-heterogeneity. Indeed, there are known examples of seemingly stochastic plastic outputs when the environmental conditions approach the so called “neutral point,” i.e., a set of conditions near the threshold zone of responsiveness (Nijhout, 1975; Emlen, 1996; West-Eberhard, 2003). In such a case, reactiveness to the environment is still robust, but only a fraction of organisms happens to experience the amount of combined inducing signal above the threshold value. However, given our current knowledge it is difficult to disentangle between the two scenarios. Therefore, additional studies are needed to demonstrate if mechanisms converting developmental noise to stochastic phenotypic output exist in *P. pacificus*. Nevertheless, the seemingly stochastic action of the switch network does not lead to the production of intermediate morphologies, corroborating our expectation that studying discontinuous plasticity allows to disentangle the robustness of the switch network and the robustness of the phenotype.

## Robustness of the Phenotype

Several mechanisms were suggested to buffer the development of both invariable and plastic traits against stochastic environmental and genetic variation. The best studied mechanism is provided by heat shock proteins of the Hsp90 family. A naive idea that chaperones maintain normal functioning of cells because they can refold proteins destabilized by weakly deleterious mutations or by environmental influences prompted a wave of experiments in various organisms, which showed that cryptic variation is indeed uncovered once the Hsp90 function is compromised (Rutherford and Lindquist, 1998; Queitsch et al., 2002; Rohner et al., 2013). Interestingly, subsequent studies provided evidence that the role of Hsp90 proteins may be more complex than simply exhibiting chaperone activity. Namely, they were implicated in the regulation of piRNA production, which in turn may silence deleterious gene variants (Gangaraju et al., 2011). Further research in yeast, animals and plants demonstrated that complementary mechanisms also exist. For example, the prion form [*PSI^+^*] of the translation release factor Sup35 in *Saccharomyces* yeasts allows stop codon readthrough and thus releases the cryptic genetic variation accumulated in the 3′ untranslated regions of genes (Masel and Griswold, 2009). In *C. elegans*, the remarkable reproducibility of the division pattern of seam cells (epidermal stem cells) is provided by the action of a basic helix-loop-helix (bHLH) transcription factor LIN-22 (Katsanos et al., 2017). In *Arabidposis*, robust timing and positioning of organs on the stem is generated by a common action of two hormone-based fields (Besnard et al., 2014). In addition to studies on single genes, simulations of complex gene networks, followed by large-scale mutant screens, demonstrated that functional knockdowns of 5% of all genes in *S. cerevisiae* decrease phenotypic robustness (Bergman and Siegal, 2003; Levy and Siegal, 2008; Bauer et al., 2015). Studies of developmental stability in recombinant inbred lines in *Arabidopsis thaliana* also unraveled multiple quantitative trait loci (QTL) associated with robustness against genetic and environmental variation (Hall et al., 2007; Fu et al., 2009). Nevertheless, heat shock proteins remain the best studied capacitors of morphological variation to date. Importantly, the emergence of aberrant phenotypes after developmental buffering by Hsp90 is alleviated was observed in laboratory populations of *Drosophila* and *Arabidopsis*, which are nearly isogenic (Rutherford and Lindquist, 1998; Queitsch et al., 2002). This indicates that not only cryptic genetic variation, but also environmental micro-heterogeneity and developmental noise are likely sources of stochastic variation buffered by heat shock proteins.

The proposed independence of the phenotypic buffering from the action of the switch suggests that the inhibition of the Hsp90 machinery in *P. pacificus* should lead to a change in the distribution of mouth morphologies, whereby the Eu and St morphs could nevertheless still be distinguishable even if distorted (**Figure 1C**). To visualize the extent of morphological differences between individuals, morphologies can be quantified using geometric morphometric analysis. As expected, in *P. pacificus* and other dimorphic species of the same nematode family, the Eu and St morphs form separate clusters in the morphospace with no overlap (Susoy et al., 2015). In accordance with the prediction, manipulation of Hsp90 activity through life-long exposure to elevated temperature, pharmacological inhibition or knockout of the Hsp90-encoding gene *daf-21* increased the morphological variation of the Eu and St morphs in *P. pacificus* (Sieriebriennikov et al., 2017) (**Figures 2E,F**). Specifically, rearing animals at the highest sublethal temperature displaced the distribution of the mouth morphologies in the morphospace, an effect that was observable in both morphs. In contrast, applying the pharmacological inhibitor of Hsp90 function induced expansion of morphology distributions without any evident shift. Finally, *daf-21*/Hsp90 knockout generated using CRISPR/Cas9 resulted in a combined effect, whereby the distribution of morphologies was displaced in the morphospace and morphological disparity was increased (**Figures 2E,F**). These observations demonstrate that the mechanism that provides developmental buffering against genetic and environmental perturbations acts to canalize the development of the discrete morphs in *P. pacificus*. Importantly, although some treated animals exhibited intermediate morphologies, most individuals could still be classified into Eu and St. Additionally, introduction of the *daf-21*/Hsp90 mutation in the Eu-constitutive *nhr-40* mutant line did not lead to the appearance of St animals, but only increased the morphological variation of the Eu morphs (Sieriebriennikov et al., 2017). This finding strongly supports the hypothesis that the two types of robustness of plastic traits described here – robustness of environmental responsiveness and robustness of phenotypic output – are provided by at least partially non-overlapping mechanisms.

## Conclusion

In summary, the *P. pacificus* mouth-form polyphenism allows two major conclusions with regard to the relationship of plasticity and robustness. First, we propose that robustness and plasticity are complementary rather than opposing phenomena. Second, we argue that knowledge about the mechanisms of plastic development enables formulation of testable hypotheses about the interplay between plasticity and robustness. Specifically, separation between the developmental switch gene network and the gene network executing the selected phenotype (**Figure 1A**) strongly suggests that plastic traits require robustness at two levels. Firstly, the switch network must generate a robust response to the multitude of environmental inputs despite developmental noise and other stochastic perturbations. Secondly, the execution network must generate a robust phenotypic outcome within a constrained range of possible phenotypes.

To demonstrate how these questions can be addressed, we use an example of a self-fertilizing nematode that exhibits a discrete plasticity of feeding structures. In *P. pacificus*, manipulation of culture conditions or introduction of mutations in the switch network only influences the ratio between the morphs and not the alternative morphologies themselves, supporting the long-standing prediction that the switch genetic network is developmentally independent from the network involved in building the morphologies. We discuss the phenomenon of apparent stochasticity of morph ratios in fixed culture conditions, which was previously suggested to be a bet-hedging strategy, and propose that it may be linked to limited robustness of the switch network to developmental noise. Importantly, such stochasticity only affects the morph ratios and not the morphology. Further, we discuss experiments in which developmental buffering by Hsp90 was compromised, which changed the distribution of morphologies of both morphs. Yet, both morphs were still produced even though their ratio was somewhat shifted. Together, these observations corroborate our hypothesis that robustness of the switch and robustness of the execution network are provided by at least partially non-overlapping mechanisms.

While our knowledge of plastic development of the feeding structures in *P. pacificus* is far from being complete, the approaches discussed here pave the way to reconcile plasticity and robustness. Both phenomena are suggested to promote evolution and more mechanistic studies are necessary to elucidate the genetic and physical basis of their interaction. Therefore, we would like to encourage similar studies in other models, which will verify our conceptualizations and provide new insight into addressing the relationship between plasticity and robustness, and their role in evolution.

## Author Contributions

BS and RS conceived and wrote the manuscript.

## Conflict of Interest Statement

The authors declare that the research was conducted in the absence of any commercial or financial relationships that could be construed as a potential conflict of interest.
